# Food Supplements Containing Iron—Comparison of Actual Content with Declared Content and Health Consequences

**DOI:** 10.3390/molecules29204796

**Published:** 2024-10-10

**Authors:** Anna Puścion-Jakubik, Klaudia Maria Zimnoch, Katarzyna Socha

**Affiliations:** Department of Bromatology, Faculty of Pharmacy with the Division of Laboratory Medicine, Medical University of Białystok, Mickiewicza 2D Street, 15-222 Białystok, Poland; kzimnoch1@student.umb.edu.pl (K.M.Z.); katarzyna.socha@umb.edu.pl (K.S.)

**Keywords:** food supplements, pharmacy, safety of supplementation, compliance with the manufacturer’s declaration

## Abstract

The use of food supplements (FSs) is becoming an increasingly common trend observed in society. This is related to frequently observed nutritional deficiencies and the inability to provide sufficient amounts of nutrients, including vitamins and minerals, through the diet. The ease of registering FSs sometimes results in low-quality preparations on the market. Therefore, our research aimed to assess the content of one of the most popular trace element components, iron (Fe), in FSs available in Poland. This study covered 109 preparations purchased from stationary pharmacies and online pharmacies. The following criteria were used to characterize FSs in the data analysis: the Fe content declared by the manufacturer, pharmaceutical form, type of Fe salt, manufacturer’s country of origin, contents of other trace elements or minerals, presence of additional ingredients, age of the target group, and addition of vitamins B2, B6, B12, and C. The Fe content was quantified using atomic absorption spectrometry after mineralization using microwaves. It was demonstrated that 69.73% of the preparations contained more Fe than the value declared by the manufacturer (and corrected for permissible deviations), 11.00% contained less Fe than declared, and only 19.27% were within the norm. In summary, the FS market requires the improvement of manufacturing processes and increased control, which should translate into patient safety.

## 1. Introduction

Supplementation is an increasingly common way of taking care of health—the use of food supplements (FSs) with various compositions is becoming a trend observed in society. It involves supplementing deficiencies in vitamins, minerals, and other substances which have different physiological effects, if it is not possible to supply sufficient amounts through the diet [1,2].

In Poland, introducing an FS to the market currently requires submitting a notification to the Chief Sanitary Inspectorate, along with the packaging design. Unlike medicines, the legislator does not require the presentation of tests confirming quality, including confirmation of the contents of the ingredients declared on the label. In other European countries, e.g., Germany, FSs are also subject to food laws, not pharmaceutical laws [3,4,5].

The Regulation of the European Parliament and of the Council of 20 December 2006 specifies 13 vitamins and 15 minerals that may be present in FSs. One of the ingredients may be iron (Fe), occurring in the following chemical forms: ferrous carbonate, ferrous citrate, ferrous ammonium citrate, ferrous gluconate, ferrous fumarate, sodium iron diphosphate, ferrous lactate, ferrous sulfate, ferric diphosphate (ferric pyrophosphate), ferrous saccharate, and elemental iron (carbonyl + electrolytic + hydrogen reduced) [6,7].

Fe performs many important functions in the human body and occurs in hemoglobin, myoglobin, tissue enzymes, and ferritin (in a stored form). The main function of Fe is related to the processes of cellular respiration. It also participates in the process of creating red blood cells in the bone marrow, as well as in the detoxification of harmful substances in the liver [1,8].

Dietary reference values (DRVs) for Fe have been identified at the request of the European Commission by the Panel on Dietetic Products, Nutrition, and Allergies. For men, the average requirement (AR) is 6 mg/day, and the population reference intake (PRI) is 11 mg/day. The same DRV applies to postmenopausal women as to men. For premenopausal women, the PRI is set at 16 mg/day due to the loss of Fe during menstruation. The requirements for children are as follows: 11 mg/day for infants aged 7 to 11 months, 7 mg/day for children aged 1 to 6 years, and 11 mg/day for children aged 7 to 11 years and boys aged 12–17 years. For girls aged 12–17 years, the PRI is 13 mg/day. For pregnant and breastfeeding women, the DRVs are the same as those for premenopausal women [8].

The above bodily requirements should be met with good-quality products. About 10–15% of Fe is usually absorbed from food. Increased absorption occurs in the case of deficiencies in the body. Moreover, better absorption occurs with the heme form, while lower absorption occurs in the presence of plant protein, polyphenols, phytates, and certain minerals in meals, such as calcium [1,8].

The quality of FSs is an extremely important element in the prevention of lifestyle diseases due to their widespread use. The assumption behind the use of FSs is that they should be used by healthy people, but patients often use these preparations when they notice the first symptoms of deficiencies.

Patients take FSs with Fe when they notice symptoms of deficiency in this trace element, such as anemia, which results in pallor of the mucous membranes, lesions in the corners of the mouth, brittle nails and hair, skin roughness, reduced physical fitness, ability to concentrate, and memory, reduced resistance to infection, etc. [1,9].

Deficiencies most often result from a small food supply, low content of digestible forms, absorption disorders, or excessive loss due to inflammation, infections, cancer, or transferrin deficiency. What is particularly dangerous is that Fe deficiency in the body may lead to increased Cd and Pb concentrations in the blood [9].

So far, no cases of Fe toxicity have been reported as it occurs naturally in food. Too high a Fe supply from pharmaceutical preparations may result in nausea, vomiting, and diarrhea. Other symptoms may occur in the circulatory, nervous, and excretory systems. It should also be emphasized that too much Fe leads to an increase in the production of free radicals and, consequently, to an increase in the risk of diseases with this pathomechanism [1,9]. However, supplementation is very common, so it is necessary to assess the safety of commercially available preparations [10].

Preparations recommended for patients with special nutritional needs, e.g., pregnant and breastfeeding women, infants and small children, and adults with known deficiencies, are available for sale. Fe is often an additive in multivitamins and mineral preparations. Therefore, our research aimed to assess the compliance of manufacturers’ declarations with the actual Fe content in FSs, as well as to characterize the market of the most popular preparations containing Fe, considering important pharmaceutical parameters such as the Fe salt used or the pharmaceutical form of the preparations.

## 2. Results

The results characterizing the Fe content in the tested FSs are presented in Table 1, Table 2, Table 3, Table 4, Table 5, Table 6, Table 7, Table 8 and Table 9.

The following criteria were adopted for the division of the FSs: the content declared on the package, the pharmaceutical form, the Fe salt, the number of minerals (in single-component and multi-component preparations, i.e., containing only Fe or Fe and at least one other trace element or mineral), and the addition of other ingredients, including B vitamins and vitamin C.

Table 1 presents the Fe content in one serving of FS, considering the content declared by the manufacturer (to simplify the statistical analyses, range criteria were adopted, whereas producers precisely declared the contents). It was shown that the median Fe content was higher in preparations with a declared content ranging from 10 to 20 than in preparations with a declared amount above 20 mg (21.96 vs. 14.99 mg/serving).

**Table 1:** The Fe content (mg) measured in one serving of food supplement compared with the Fe content declared by the manufacturer.

Declared Content (Group—Signage)	*n*	Fe Content (mg/Serving)				
		Av. ± SD	Min.–Max.	Med. (Significance)	**Q1**	**Q3**
Less than 10 mg (1)	58	7.89 ± 5.00	0.91–24.54	6.88 (**^1/2,^ *^1/3^)	4.72	9.51
10–20 mg (2)	41	21.83 ± 9.97	>dl–56.61	21.96 (**^1/2^)	15.56	26.00
Above 20 mg (3)	10	19.71 ± 17.04	3.43–52.28	14.99 (*^1/3^)	5.41	34.76
Total	109	14.22 ± 10.97	>dl–56.61	10.83	6.44	21.66

Av.—average, dl—detection limit, Max.—maximum value, Med.—median, Min.—minimum value, Q1—lower quartile, Q3—upper quartile, SD—standard deviation, * *p* < 0.05, and ** *p* < 0.001.

Table 2 presents the Fe contents depending on the pharmaceutical form. The highest median content was recorded for effervescent tablets (14.70 mg/serving), while the lowest was recorded for liquids (3.85 mg/kg).

**Table 2:** Determined Fe content (mg) per serving of food supplement considering their pharmaceutical form.

Pharmaceutical Form	*n*	Fe Content (mg/Serving)				
		Av. ± SD	Min.–Max.	Med.	Q1	Q3
Capsule	26	15.63 ± 12.64	1.55–52.28	13.49	6.48	21.90
Effervescent tablet	11	15.87 ± 7.31	6.44–26.00	14.70	8.30	22.12
Liquid	4	9.03 ± 13.08	>dl–28.40	3.85	1.34	16.71
Powder	4	14.08 ± 5.81	9.00–20.42	12.82	9.00	20.42
Tablet	60	14.35 ± 11.16	0.91–56.61	10.56	6.43	21.81
Other	5	5.90 ± 1.71	4.24–8.52	5.41	4.72	6.62

Av.—average, dl—detection limit, Max.—maximum value, Med.—median, Min.—minimum value, Q1—lower quartile, Q3—upper quartile, and SD—standard deviation.

Table 3 presents the Fe contents in the tested FSs depending on the Fe salt contained in the preparation. The highest median was recorded for preparations containing iron diphosphate (28.39 mg/serving), while the lowest was recorded for iron (II) gluconate (5.54 mg/serving). It should be emphasized that not all chemical forms of Fe present in the preparations are included in the list of acceptable ones—for example, iron (II) bis-glycinate.

**Table 3:** Fe content (mg) in one serving of food supplement depending on the Fe salt compound.

Type of Fe Salt Compound	*n*	Fe Content (mg/Serving)				
		Av. ± SD	Min.–Max.	Med.	Q1	Q3
Iron (II) fumarate	39	16.72 ± 12.18	0.98–56.61	12.58	6.78	25.52
Iron (II) gluconate	12	7.65 ± 8.13	0.91–26.00	5.54	2.17	8.46
Iron (II) lactate	9	7.89 ± 4.79	2.68–17.66	6.82	5.02	8.81
Iron (II) sulfate	11	16.75 ± 7.47	4.24–26.06	18.11	11.36	23.07
Iron diphosphate	3	33.70 ± 16.58	20.41–52.28	28.39	20.42	52.28
Iron (II) bis-glycinate	17	14.74 ± 10.50	1.55–41.33	14.40	7.57	21.66
Iron (III) pyrophosphate	4	8.89 ± 4.62	4.40–12.94	9.11	4.90	12.88
Elemental iron	4	8.80 ± 9.74	<dl–15.88	6.53	1.72	15.88
Other	4	15.03 ± 10.94	5.41–30.19	12.26	7.20	22.86
No specific form	6	11.40 ± 7.25	1.45–23.63	10.14	9.32	13.75

Av.—average, dl—detection limit, Max.—maximum value, Med.—median, Min.—minimum value, Q1—lower quartile, Q3—upper quartile, and SD—standard deviation.

Pharmaceutical preparations containing only one mineral (or trace element) were characterized by a higher median Fe content, but these differences were not statistically significant (13.64 vs. 9.51 mg/serving) (Table 4).

**Table 4:** Fe content (mg) in a serving of food supplement depending on the amount of minerals or trace elements.

Amount of Minerals	*n*	Fe Content (mg/Serving)				
		Av. ± SD	Min.–Max.	Med.	Q1	Q3
Only Fe	34	16.71 ± 14.08	<dl–56.61	13.64	5.41	21.92
Multimineral preparations	75	13.09 ± 9.12	0.91–42.85	9.51	6.56	21.53

Av.—average, dl—detection limit, Max.—maximum value, Med.—median, Min.—minimum value, Q1—lower quartile, Q3—upper quartile, and SD—standard deviation.

We also show that preparations produced in Poland contained a higher amount of Fe ions per serving, but these were not significantly different compared with preparations produced in other countries (11.91 vs. 8.72 mg/serving—Table 5).

**Table 5:** Fe content (mg) in a serving of food supplement depending on the country of origin of the manufacturer.

Origin of Manufacturers	*n*	Fe Content (mg/Serving)				
		Av. ± SD	Min.–Max.	Med.	Q1	Q3
Poland	95	14.69 ± 11.39	<dl–56.61	11.91	6.44	21.96
Foreign manufacturer	14	11.00 ± 7.10	2.97–26.37	8.72	6.30	17.06

Av.—average, dl—detection limit, Max.—maximum value, Med.—median, Min.—minimum value, Q1—lower quartile, Q3—upper quartile, and SD—standard deviation.

We also noted that preparations recommended for children had a higher Fe content than preparations intended only for adults (14.17 vs. 10.29 mg/serving), as presented in Table 6.

**Table 6:** Fe content (mg) in a serving of food supplement depending on the age group for which the food supplement is recommended.

Age Category	*n*	Fe Content (mg/Serving)				
		Av. ± SD	Min.–Max.	Med.	Q1	Q3
Adults	95	14.22 ± 11.29	<dl–56.51	10.29	6.30	21.92
Kids	14	14.25 ± 8.85	2.68–30.19	14.17	6.62	20.42

Av.—average, dl—detection limit, Max.—maximum value, Med.—median, Min.—minimum value, Q1—lower quartile, Q3—upper quartile, and SD—standard deviation.

Preparations containing only Fe (group: no additives) had the highest median Fe content (17.41 mg/serving), but these values were not statistically significant compared to preparations containing different categories of additives. High values were also recorded for FS-containing vitamins (15.53 mg/serving) and vitamins and minerals (13.81 mg/serving). These data are presented in Table 7.

**Table 7:** Fe content (mg) in a serving of food supplement depending on other ingredients in the preparations.

Group of Ingredients	*n*	Fe Content (mg/Serving)				
		Av. ± SD	Min.–Max.	Med.	Q1	Q3
Vitamins	23	19.58 ± 15.28	2.68–56.61	15.53	6.44	28.31
Vitamins and minerals	34	15.60 ± 9.25	2.97–42.85	13.81	8.22	23.07
Vitamins, minerals, and ingredients of bee origin	4	7.87 ± 6.62	1.23–16.61	6.81	2.98	12.76
Vitamins, minerals, and plant ingredients	37	11.35 ± 8.78	0.91–37.16	9.32	6.11	13.75
Vitamins and raw plant materials	5	5.04 ± 2.98	2.32–9.63	5.41	2.45	5.42
No additives	6	15.44 ± 9.85	<dl–28.40	17.41	9.00	20.42

Av.—average, dl—detection limit, Max.—maximum value, Med.—median, Min.—minimum value, Q1—lower quartile, Q3—upper quartile, and SD—standard deviation.

We noted that the content of selected B vitamins, such as vitamin B2, B9, and B12, as well as vitamin C, did not affect the Fe content (Table 8).

**Table 8:** Fe content (mg) in a serving of food supplement depending on the presence of additional ingredients.

Content of Selected Ingredients		*n*	Fe Content (mg/Serving)				
			Av. ± SD	Min.–Max.	Med.	Q1	Q3
Vitamin B2	No	41	16.29 ± 14.06	<dl–56.61	14.40	5.41	21.92
	Yes	68	12.97 ± 8.48	1.23–37.16	9.48	6.59	21.01
Vitamin B9	No	34	11.13 ± 10.43	<dl–42.85	8.66	2.97	13.75
	Yes	75	15.62 ± 11.00	1.23–56.61	12.94	6.78	22.12
Vitamin B12	No	31	13.19 ± 10.56	<dl–41.33	11.91	5.03	20.42
	Yes	78	14.63 ± 11.17	10.98–56.61	10.56	6.73	21.92
Vitamin C	No	17	12.89 ± 8.80	<dl–28.76	11.91	6.30	19.00
	Yes	92	14.47 ± 11.35	0.98–56.61	10.56	6.46	21.91

Av.—average, dl—detection limit, Max.—maximum value, Med.—median, Min.—minimum value, Q1—lower quartile, Q3—upper quartile, and SD—standard deviation.

Table 9 presents a summary of our research results in relation to the recommendations, indicating the acceptable ranges of deviations for the mineral content of food supplements. Notably, 70% of the tested preparations contained more Fe than the declared value and the recommendations (i.e., at least 45% more than the declaration), 11% of the preparations contained less Fe, while only 19% of FSs on the market met applicable standards.

**Table 9:** Percentage of food supplements with the Fe content within the normal range and below and above the declared value according to the analyzed factors (*p* > 0.05).

Criterion	Subgroup	Below Standard *n* (%)	Normal *n* (%)	Above Normal *n* (%)
Declared content	Less than 10 mg	0 (0.00)	11 (10.09)	47 (43.12)
	10–20 mg	5 (4.59)	9 (8.26)	27 (24.77)
	Above 20 mg	1 (0.92)	7 (6.42)	2 (1.83)
Form	Capsule	5 (4.59)	3 (2.75)	18 (16.51)
	Liquid	1 (0.92)	0 (0.00)	3 (2.75)
	Powder	0 (0.00)	1 (0.92)	2 (1.83)
	Effervescent tablet	0 (0.00)	7 (6.42)	4 (3.67)
	Tablet	5 (4.59)	10 (9.17)	45 (41.29)
	Other	1 (0.92)	0 (0.00)	4 (3.67)
Fe salt	Iron (II) fumarate	1 (0.92)	8 (7.34)	30 (27.52)
	Iron (II) gluconate	1 (0.92)	5 (4.59)	6 (5.50)
	Iron (II) lactate	0 (0.00)	2 (1.83)	7 (6.42)
	Iron (II) sulfate	1 (0.92)	1 (0.92)	9 (8.26)
	Iron diphosphate	0 (0.00)	0 (0.00)	3 (2.75)
	Iron (II) bis-glycinate	4 (3.67)	2 (1.83)	11 (10.09)
	Iron (III) pyrophosphate	0 (0.00)	1 (0.92)	3 (2.75)
	Elemental iron	2 (1.83)	1 (0.92)	1 (0.92)
	Other	2 (1.83)	1 (0.92)	1 (0.92)
	No specific form	1 (0.92)	0 (0.00)	5 (4.59)
Amount of minerals/trace elements	Only Fe	10 (9.17)	7 (6.42)	17 (15.60)
	Multimineral preparations	2 (1.83)	14 (12.84)	59 (54.14)
Origin of manufacturers	Poland	12 (11.00)	20 (18.35)	63 (57.81)
	Foreign manufacturer	0 (0.00)	1 (0.92)	13 (11.92)
Age group	Adults	11 (10.09)	19 (17.43)	65 (59.64)
	Kids	1 (0.92)	2 (1.83)	11 (10.09)
Groups of ingredients	Vitamins	7 (6.42)	4 (3.67)	12 (11.00)
	Vitamins and minerals	0 (0.00)	8 (7.34)	26 (23.86)
	Vitamins, minerals, and ingredients of bee origin	1 (0.92)	2 (1.83)	1 (0.92)
	Vitamins, minerals, and plant ingredients	1 (0.92)	4 (3.67)	32 (29.37)
	Vitamins and raw plant materials	1 (0.92)	1 (0.92)	3 (2.75)
	No additives	2 (1.83)	2 (1.83)	2 (1.83)
Content of vitamin B2	No	10 (9.17)	10 (9.17)	21 (19.27)
	Yes	2 (1.83)	11 (10.09)	55 (50.47)
Content of vitamin B9	No	5 (4.59)	7 (6.42)	22 (20.18)
	Yes	7 (6.42)	14 (12.84)	54 (49.55)
Content of vitamin B12	No	8 (7.34)	6 (5.50)	17 (15.60)
	Yes	4 (3.67)	15 (13.76)	59 (54.13)
Content of vitamin C	No	5 (4.59)	5 (4.59)	7 (6.42)
	Yes	7 (6.42)	16 (14.68)	69 (63.30)
Total		12 (11.00)	21 (19.27)	76 (69.73)

According to the Resolution of the Team for Dietary Supplements, the maximum allowable content of Fe in an FS is 20 mg, except for FSs recommended for pregnant women, wherein the allowable content is 30 mg [11].

Of all the FSs tested, 6.42% contained more than 30 mg of Fe (only one preparation had a word suggesting motherhood in its name), while 29.36% contained more than 20 mg per serving.

**Figure 1:**
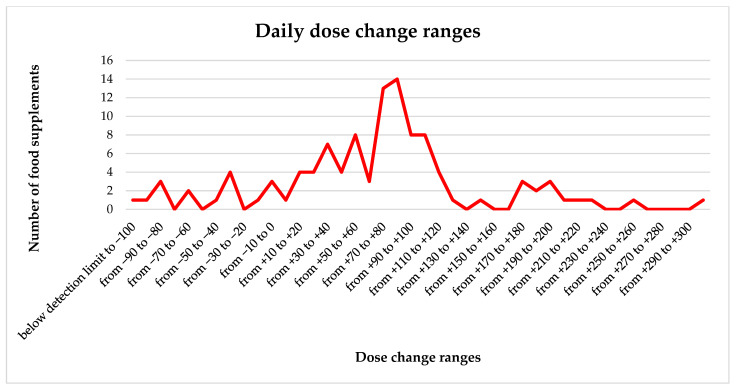
The number of preparations in terms of percentage changes in the adopted daily dose of Fe, taking into account the results obtained in this publication.

Figure 1 presents the actual values of the deviations; it indicates how much more or less Fe the patient takes per day (taking into account the manufacturer’s recommendations regarding dosage) compared to the declaration (i.e., the value specified on the packaging, expected by the patient), and how many samples of preparations fall into each range of deviations. For example, the Fe values of as many as 14 preparations ranged from 80 to 90% more than the declaration on the packaging.

**Figure 2:**
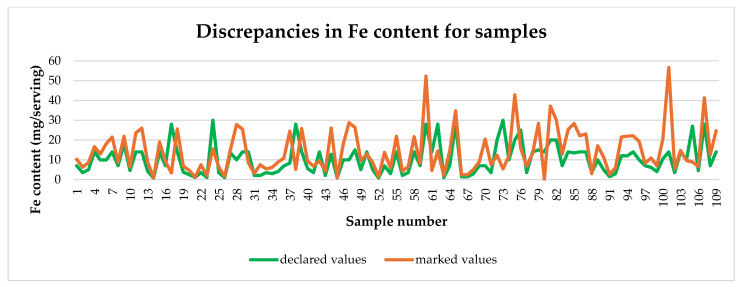
Comparison of declared Fe content with actual content.

Figure 2 presents detailed results for all tested dietary supplements. For example, the highest determined Fe content was 56.61 mg in one serving, while the declaration indicated 14 mg.

## 3. Discussion

Our research shows that almost 70% of FSs available for sale contained more than 145% of the Fe content declared by the manufacturers (100% means that the declared and marked values are completely consistent, and 45% represents an acceptable spread). The topic of comparing the declared values of trace elements or minerals with the values determined using analytical methods is becoming more and more popular. The topic of the quality of FSs containing minerals or trace elements has been discussed by our team in terms of the content of magnesium (Mg) [12] and calcium (Ca) [13]. We showed significant deviations from the values declared on the packaging. In the case of Mg, a large percentage of preparations contained less than the declared values, and the opposite tendency was observed in the case of Ca.

Pawlak et al. (2016) [14] determined the Fe contents in 20 vitamin and mineral supplements from Poland. The conclusions drawn by the authors were similar to ours: in most of the tested preparations, the determined Fe content differed from the declared one, and the difference between the determined and declared contents of this mineral did not depend on factors such as price, form, and Fe content. The authors showed that the content deviated beyond the permissible limits in only one preparation, wherein it was 64% higher than the declared value. Our results, however, are more concerning, estimating a five-fold higher amount of Fe, and as many as 69.73% of the tested supplements contained more Fe than allowed.

Another study [15] including 29 preparations assessed the compliance of the declared values with the actual contents. The Fe content was determined using the AAS method. The authors showed that 14% (that is, four FSs) did not meet European requirements regarding acceptable ingredient tolerance limits. For 52% of supplements, the determined Fe content was higher than the declared value, and for 48%, it was lower. The range of variability was from −83% to +56%.

Another publication [16] assessed the quality of 18 FSs available in Palestine. FSs came from local as well as international producers. The Fe content was determined via potentiometric titration and AAS. Two analysis methods were used because according to the International Pharmacopoeia, a sample is considered positive if the analysis result is within the acceptable range. If the result was discrepant, the Fe content was assessed using a second method. If the two analyses yielded discrepant results, the sample did not meet Good Laboratory Practice (GLP) requirements. It was estimated that 72.22% of the samples did not meet acceptable standards. The samples of inappropriate quality included four preparations in the form of tablets, four in the form of a solution, three in the form of hard capsules, and two in the form of soft capsules.

Studies [17] assessing the quality of dietary iron supplements were also carried out in Libya, where eight preparations were selected. The Fe content was assessed using a method based on spectrophotometric measurement of the Fe content after complexation with 1,10-phenanthroline in an acidic environment. The authors showed an average content of 60 mg Fe/tablet (minimum value: 40.07 mg; maximum value: 112.63 mg). It was estimated that as many as 75% of the preparations had a lower Fe content than the value indicated by the manufacturer.

The available publications did not always focus on comparing the declared and actual values. Sometimes, the authors’ goal was to determine the content and assess whether these preparations could be a supplement to daily food rations. For example, the publication by Błoniarz and Zaręba (2007) [18] examined the content of Fe in five FSs used to reduce body weight. The determined amounts were 0.00276 ± 0.00067 mg/serving, 0.0256 ± 0.00419 mg/serving, 0.0626 ± 0.0131 mg/serving, 1.254 ± 0.0821 mg/serving, and 0.0819 ± 0.0472 mg/serving, which raises the question of the validity of supplementation with preparations containing, sometimes, very low doses of Fe.

Such large discrepancies in Fe content may be caused by several factors. One of the reasons may be the production processes, during which the degradation of components may occur, among others, as a result of pharmaceutical interactions, which is related to their durability. The quality of the final product also depends on the quality of individual components, including excipients. In addition, preparations sold in pharmacies are subject to the controlling of storage conditions (including temperature), so it seems necessary for manufacturers to monitor both storage conditions and transport conditions, similarly to what is required for medicines. Another significant problem is EU regulations, which are not very restrictive and allow for a large positive deviation in the content of mineral components; this is a particularly important issue in the case of minerals that show toxicity during long-term supplementation with high doses. Polish legislation is subject to European restrictions on this matter. Tightening these regulations seems to be difficult to implement due to the enormous development of this market, which translates into high sales and high profits for producers.

The consequences of inappropriate Fe content in a preparation, especially when patients chronically use the same preparation with content different than the declared one, can be two-way in nature. The first category, resulting from too low a content, is associated with a permanent deficiency. The deficiency causes characteristic symptoms mentioned earlier in the introduction, such as skin symptoms or disorders of the nervous system. The second category, resulting from too high a supply, also includes health effects such as hemochromatosis or increased oxidative stress. Hemochromatosis is a disease caused by excessive absorption of iron from the digestive tract. It can have primary, i.e., hereditary, secondary, and mixed forms. Secondary and mixed forms are caused by excessive Fe supply or reduced iron consumption in disorders of the circulatory system. One of the symptoms is increased skin pigmentation, which is accompanied by non-specific symptoms such as chronic fatigue or joint pain [19]. Fe not absorbed in the large intestine causes irritation of gastrointestinal epithelial cells, promoting oxidative stress via the Fenton reaction. In addition, an excess supply is indicated to promote oxidative stress in the systemic circulation, which has been demonstrated in different populations [20].

This study has several limitations. The results obtained indicate a large discrepancy between the declared values and the actual contents, which raises concerns that other batches of the same supplements may contain contents that are even more different. The intention of this study was to select the most representative samples; i.e., the most popular dietary supplements were selected. Therefore, the least popular preparations were not included in this project. This study was conducted on preparations available in Poland, and therefore, FSs available for sale in other countries may differ in content, which is due to national standards and control systems.

Future studies should focus on assessing the content of other trace elements or minerals in FSs to ensure the efficacy and safety of supplementation for patients, especially in groups susceptible to side effects. In addition, it is necessary to assess the method of releasing Fe from different combinations and determine the factors influencing this process, such as the content of other minerals and the addition of B vitamins and vitamin C, but also the presence of components in the diet that hinder absorption (such as calcium, phytates, or polyphenols). The pharmaceutical form of the preparation or the form of Fe contained in FSs is also important because they differ in bioavailability, but also in patient tolerance—in the case of troublesome gastrointestinal complaints, patients may give up using the preparations. It is also important to consider whether there are pharmaceutical interactions between the components, which may translate into the actual content of the tested component.

## 4. Materials and Methods

### 4.1. Materials

Samples of FSs were chosen for analysis based on the results of our survey research [10] and considering the popularity rankings of FSs in selected pharmacy chains in Poland.

Other inclusion criteria were the registration of the preparation by the manufacturer as an “FS” and being within the expiration date.

In this study, the analysis covered 109 different FSs that were purchased from online and stationary pharmacies in 2022–2023. Trade names are not disclosed because our goal was to statistically assess the quality of FSs containing Fe, and not to indicate irregularities in specific preparations—this is the task of control authorities.

This study included preparations with different declared Fe contents, pharmaceutical forms, and countries of origin of the manufacturers, containing one (Fe) or several minerals or trace elements, as well as ingredients increasing absorption of Fe.

Samples for testing were taken from different blisters of tablets or other solid pharmaceutical forms, with 10 subsamples taken for each, and a collective sample was created. Analyses were performed in three repetitions.

The main claims included on the labels of the tested FSs were as follows: Fe affects proper cognitive functions in children, supports the formation of hemoglobin and red blood cells, supports the proper functioning of the immune system, supports proper oxygen transport, and contributes to the reduction of fatigue and weariness.

The preparations were recommended, among others, to blood donors, people with increased physical activity, vegetarians, people with an increased need for Fe, and to women who are planning a pregnancy, menstruating heavily, pregnant, and breastfeeding.

In addition, FS packaging contained standard information on safety precautions, including the following: do not use if you are hypersensitive to any ingredient of the product; do not exceed the recommended daily portion of the product; a balanced diet and a healthy lifestyle are important for maintaining health; the dietary supplement cannot be used as a substitute (replacement) for a varied diet; the preparations should be stored at room temperature, out of the reach of children; and during pregnancy and breastfeeding, consult a doctor before use.

### 4.2. Sample Preparations

The FS samples were homogenized in a vibratory mill (Testchem, Rybnicka, Poland) and then weighed to approximately 0.3 g (with an accuracy of 1 mg) into mineralization vessels. Next, 4 mL of spectrally pure 69% nitric acid (Tracepur, Merck, Darmstadt, Germany) was added. The wet microwave digestion procedure was conducted in a closed system (mineralizer: Berghof, Speedwave, Eningen, Germany), as shown in Table 10. Initially, mild conditions were used for FS mineralization due to the higher content of organic matter. As the process progressed, the values of individual parameters (temperature, pressure, microwave power) increased to obtain the best possible sample dissolution and decomposition into inorganic compounds.

Then, the mineralizates were quantitatively transferred to vessels using deionized water.

### 4.3. Determination of the Fe Content

The Fe concentration was measured using atomic absorption spectrometry (AAS) in an acetylene–air flame at a wavelength of 248.3 nm with Zeeman background correction. A calibration curve was created based on the dependence of the absorbance on the concentration, from which the Fe content was read. The curve range is 5 mg/L—the mineralizates were appropriately diluted before determination. The detection limit, expressed as a characteristic concentration, was 0.19 mg/kg.

### 4.4. Validation of the AAS Method

To control the accuracy of the analyses performed, a certified reference material (CRM) was used (Simulated Diet D, LIVSMEDELS VERKET, National Food Administration, Sweden). All obtained values, determined before analysis for every 10 samples, fell within the value range of 183–215 mg/kg, and the average was 199 ± 16 mg/kg. The coefficient of variation was V = 2.55%, and the accuracy (% of error) was 0.23%.

### 4.5. Comparison of Results with Recommendations

Guidelines for the quality of FSs indicate that the tolerance value for the content of minerals specified on the label, compared with the actual content, may range from −20 to +45% for the value declared by the manufacturer [21].

### 4.6. Statistical Analyses

The Statistica program (Tibco, Palo-Alto, CA, USA) was used to conduct statistical analyses. Basic statistics were calculated, such as means (Av.) with standard deviation (SD), minimum (Min.), and maximum (Max.) values, medians (Med.), lower quartiles (Q1), and upper quartiles (Q3). The Mann–Whitney U test was performed to show differences in the Fe content between the 2 groups, and the Kruskal–Wallis test was used for comparisons among more than 2 groups. A significance level of *p* < 0.05 was used.

## 5. Conclusions

Studies assessing the iron content in food supplements showed that the values declared by manufacturers and the actual values obtained using analytical methods differed for most food supplements. It was estimated that only 19.27% of the tested preparations were within the tolerance limits for minerals and were consistent with the guidelines. The quality of food supplements should be subject to greater control, resulting in greater patient safety.

## Figures and Tables

**Table 10:** Characteristics of the mineralization process of the food supplements.

Step	Programmed Temperature (°C)	Time (minutes)	Maximum Pressure (atm.)	Maximum Microwave Power (%)
1	170	10	20	80
2	190	10	30	90
3	210	10	40	90
4	50	18	40	0

## Data Availability

The original contributions presented in the study are included in the article, further inquiries can be directed to the corresponding author.

## References

[1] Jarosz M., Rychlik E., Stoś K., Charzewska J. (2020). Nutrition Standards for the Polish Population and Their Application.

[2] Journal of Laws 2006 no. 171 Item 1225, the Act of August 25, 2006 on Food and Nutrition Safety. https://isap.sejm.gov.pl/isap.nsf/DocDetails.xsp?id=wdu20061711225.

[3] Suplindex. https://suplindex.com/wp-content/uploads/2017/10/RAPORT-Suplementy-diety-30.08.2017.pdf.

[4] Journal of Laws of 2010 No. 91, Item 596: Regulation of the Minister of Health of 18 May 2010 Amending the Regulation on the Composition and Labeling of Dietary Supplements. https://isap.sejm.gov.pl/isap.nsf/DocDetails.xsp?id=WDU20100910596.

[5] (2012). Commission Regulation (EU) No 231/2012 of 9 March 2012 laying down specifications for food additives listed in Annexes II and III to Regulation (EC) No 1333/2008 of the European Parliament and of the Council Text with EEA relevance.

[6] (2012). Regulation (EC) No 1925/2006 Of The European Parliament And Of The Council of 20 December 2006 on the addition of vitamins and minerals and of certain other substances to foods.

[7] (2015). EFSA Panel on Dietetic Products, Nutrition and Allergies (NDA), Scientific opinion on Dietary Reference Values for iron. EFSA J..

[8] Journal of Laws 2007, Number 196, item 1425, Regulation of the Minister of Health of October 9, 2007 on the composition and labeling of dietary supplements [Dz. U. 2007, numer 196, poz. 1425.

[9] Dutt S., Hamza I, Bartnikas T.B. (2022). Molecular Mechanisms of Iron and Heme Metabolism. Annu. Rev. Nutr..

[10] Puścion-Jakubik A., Kus K., Socha K. (2021). Medical university students’ perspective on marketing of dietary supplements. Acta Pol. Pharm..

[11] Resolution No. 20/2019 of the Team for Dietary Supplements of December 13, 2019 on expressing an opinion on the maximum dose of iron in the recommended daily serving in dietary supplements.

[12] Puścion-Jakubik A., Bartosiewicz N., Socha K. (2021). Is the Magnesium content in food supplements consistent with the manufacturers’ declarations?. Nutrients.

[13] Puścion-Jakubik A., Staniaszek G., Brzozowska P., Socha K. (2022). Quality of calcium food supplements: Evaluation compared to manufacturers’ declarations. Molecules.

[14] Pawlak A., Rajczykowski K., Loska K., Ahnert B., Wiechuła D. (2016). Ocena zawartości żelaza w witaminowo—Mineralnych suplementach diety [Assessment of iron content in vitamin and mineral dietary supplements]. Bromat Chem. Toksykol..

[15] Surowiecka J., Olczyk P., Ivanova D., Kiselova-Kaneva Y., Komosinska-Vasse K. (2020). Analysis of iron content in food supplements in relations to the safety of their use. Acta Pol. Pharm..

[16] Abualhasan M., Dwaikat S., Ataya R., Ali A., Al-Atrash M. (2021). Quality evaluation of iron-containing food supplements in the Palestinian market. Food Sci. Technol. Camp..

[17] Issa R.A.M., Beshlou E., AlHanash H.B., Salem N.S.A., Almoudi F.A., Benamer M.A.A. (2020). Spectrophotometric determination of iron in dietary supplements in Libyan market. Acad. J. Res. Sci. Publ..

[18] Błoniarz J., Zaręba S. (2007). Selected microelements (Cr, Zn, Cu, Mn, Fe, Ni) in slimming preparations. Rocz. Panstw. Zakl. Hig..

[19] Kowdley K.V., Modi N.B., Peltekian K., Vierling J.M., Ferris C., Valone F.H., Gupta S. (2023). Rusfertide for the treatment of iron overload in HFE-related haemochromatosis: An open-label, multicentre, proof-of-concept phase 2 trial. Lancet Gastroenterol. Hepatol..

[20] Babayev M., Klaunig J., Silveyra P., Henschel B., Gletsu-Miller N. (2023). Impact on oxidative stress of oral, high-dose, iron supplementation for management of iron deficiency after bariatric surgery, a preliminary study. J. Trace Elem. Med. Biol..

[21] European Commission, Directorate-General for Health and Consumers December 2012 Guidance on Setting Tolerance Limits for Labeled Nutrients. https://foodsupplementseurope.org/wp-content/themes/fse-theme/documents/publications-and-guidelines/fse-setting-of-tolerances-for-nutrient-values-declared-on-a-label.pdf.

